# Evaluation and management of orthostatic headache in hypermobility disorders

**DOI:** 10.3389/fneur.2023.1321350

**Published:** 2023-12-14

**Authors:** Olga P. Fermo

**Affiliations:** Department of Neurology, Mayo Clinic Florida, Jacksonville, FL, United States

**Keywords:** orthostatic headache, hypermobility, cervicogenic, cervical instability, spontaneous intracranial hypotension, postural orthostatic tachycardia syndrome

## Abstract

Headache is a frequent symptom among patients with hypermobility spectrum disorders. This mini review focuses specifically on a challenging aspect of headache evaluation in all patients, but especially those with hypermobility – the orthostatic headache. While the differential for an orthostatic headache is overall limited, patients with hypermobility disorders have risk factors for all of the most commonly encountered orthostatic headache disorders. The most common conditions to produce orthostatic headaches are discussed – spontaneous intracranial hypotension, cervicogenic headache, and postural orthostatic tachycardia syndrome. Less common etiologies of orthostatic headache pertinent to any patient are presented in table format.

## Introduction

Headache is one of the most common symptoms in the world, and it is particular prevelant amongst those with hypermobility disorders, affecting 86% of patients with hypermobile Ehlers-Danlos-Syndrome in one study (1). While most headaches experienced by patients with hypermobility are migraines (1), which are fairly easy to diagnose, the orthostatic headache poses a diagnostic dilemma. An orthostatic headache is defined as a headache that worsens in the upright posture and is relieved by recumbency (2). It needs to be carefully (clinically) distinguished from migraine, which worsens with physical activity but not posture *per se* (3). Orthostatic headaches are typically caused by spontaneous intracranial hypotension (SIH), cervicogenic mechanisms, and postural orthostatic tachycardia (POTS). Hypermobility is a shared risk factor for all 3 causes, and each may present with overlapping symptoms and “normal” imaging. This mini-review presents the defintion, mechanism of pain, link with hypermobility, clinically distinguishable presentations, diagnostic steps and treatments for the most common causes of orthostatic headache in hypermobility disorders. Less common etiologies of orthostatic headache pertinent to any patient are presented in table format (Table 1).

**Table 1:** Differential diagnosis of orthostatic headache.

Reported conditions	Postulated mechanism(s) of pain
SIH	Traction of pain sensitive structures due to “brain sag” Intracranial venous distention Traction, disortion, or compression of cranial nerves and/or cervical nerve roots (6).
Cervicogenic headache	Axial loading of pain sensitive structures (21). Promotion of abnormal posture (22).
POTS	Relative CSF hypovolemia (due to general hypovolemia; reduced spinal venous pressure due to dependent pooling of blood upon standing) Orthostatic ischemia of the posterior head/neck musculature (23).
PPPD	Altered postural control (29). Altered sensory processing (29, 30)
Cerebrospinal fluid shunt overdrainage	Induced by: Improper pressure gradient setting Siphoning effect (31). Flight induced (32). Cerebral venous overdrainage (33).
Orthostatic hypotension	Orthostatic ischemia of the posterior head/neck musculature (23, 34) Epidural hypotension (34) Vagal dysfunction (34)
Adrenal insufficiency	Indirect (via orthostatic hypotension) (24)
Iatrogenic CSF leak	Presumed to be the same as SIH
Platypnea orthodeoxia synrome	Orthostatic hypoxia caused by a position dependent right to left shunt (35).
Syndrome of the trephined	The brain becomes susceptible to atmospheric pressure (acting on the skull defect), in the vertical position (36).
Suboccipital craniectomy	Post-surgical posterior fossa scarring leads to changes in caudal spinal dural compliance Altered skull-dura relatonship leads to sensitization of mechanosensitive pain reptors (2).
Cerebral venous thrombosis (paradoxical)	Decreased intracranial CSF volume, secondary to increased intracranial blood volume (37).
Cerebellar hemorrhage	Mass effect and valve-like impaction of cerebellum in the foramen magnum (38).
Theoretical condition	Proposed mechanism of pain
Increased compliance of the lower spinal CSF space	Decreased intracranial pressure causes compensatory, painful venous enorgement (39).

SIH, spontaneous intracranial hypotension; POTS, postural orthostatic tachycardia syndrome; PPPD, persistent postural perceptual dizziness; CSF, cerebrospinal fluid.

## Discussion

### Spontaneous intracranial hypotension

The term orthostatic headache is nearly synonymous with SIH, and while not always correct (4), an orthostatic headache is indeed the most common symptom of SIH (5–7). Most cases of SIH result from spontaneous cerebrospinal fluid (CSF) leaks in the spine. Leaks occur at sites of pre-existing dural weakness caused by dural ectasias and nerve root sleeve meningeal diverticula, adjacent to osteophytes especially in the ventral thoracic spine, or via CSF-venous fistulas (which are typically associated with meningeal diverticula). A trivial trauma such as lifting or coughing may be associated with the onset of a leak (6). Any patient with dural ectasia, meningeal diverticula (Tarlov cysts, perineurial cysts), or sharp ostephytes can develop a CSF leak, but these risk factors are in particular common in patients with connective tissue disoders (8, 9).

Spinal CSF leaks cause the characteristic orthostatic headache due to the relationship of the leak to the zero-pressure point in the spine. The zero-pressure point is located in the cervical spine. This is the point at which the CSF pressure transitions from negative (intracranially) to positive (in the spine) in the upright position. Leaks caudal to the zero-pressure point will be promoted to leak in the patient’s upright position in the face of a positive spinal pressure, creating an even lower than normal intracranial pressure when upright. The recumbent position reduces the spinal CSF pressure, equalizes the CSF pressure amongst the cranial and spinal compartment, in turn reducing the spinal leak and improving symptoms (7). Several mechanisms of head pain are implicated. A consequence of the leak below the zero-pressure point in the spine is a vacuum-like downward traction on the brain. Traction on pain-sensitive intracranial structures including the dura, venous sinuses, and cranial nerves produces pain. Not all patients with SIH manifest brain sag, and it is thought that compensatory venous distention involving the dura, venous sinuses, and around the cranial nerves also causes pain (6).

The incidence of SIH is estimated at 5 per 100,000 or 0.005% of the population (7). With fragile, at-risk, dura being nearly a sine-qua-non for SIH, it is no surprise that this syndrome is linked to hypermobility spectrum disorders. The exact prevalence of SIH in hypermobility spectrum disorders is unknown, but in prospective SIH studies, between 16 to 38% of patients showed features of Marfan syndrome, EDS, or joint hypermobility (10, 11).

The presentation of an orthostatic headache should almost always warrant exclusion of SIH. The orthostatic headache of SIH may be absent or very mild upon waking, but with prolonged time upright (2 h) starts or intensifies, being relieved by recumbency. It is not unusual for the headache syndrome to begin as a new daily persistent headache, or a thunderclap onset headache followed by new daily orthostatic headache (12). However, the headaches of SIH are not always orthostatic and may actually be absent completely. They may be purely exertional, valsalva-induced, or paradoxical headaches worse in recumbency, mimicking so called high pressure headaches (6). The presence of any of these headache types at any point in the history of an orthostatic headache should further the suspicion for SIH. Other common symptoms include but are not limited to neck pain or stiffness, nausea, dizziness, tinnitus, muffled or distorted hearing, and diplopia, and are also typically orthostatic or occuring in the second-half of the day (6). This clinical history warrants an MRI of the brain with contrast to look for the typical stigmata of intracranial hypotension (pachymeningeal enhancement, venous sinus distension, pituitary engorgement, sagging brainstem, and cerebellar tonsillar descent). If possible, an MRI of the entire spine should be done (contrast is not necessary) to either visualize the leak (if a spinal epidural fluid collection is present) or evaluate the risk factors for leak formation (i.e., spondylosis, meningeal diverticula). A meta-analysis found that up to 19% of patients with SIH have normal (absent) brain MRI findings (13). If clinical suspicion remains high, especially higher than other differential possibilities, non-targeted high volume lumbar epidural blood patching can be performed. Patients who do not respond to non-targeted blood patching at all (and the dignosis is still considered), or who respond to patching only temporarily are then generally considered for myelography for CSF leak localization to plan targeted treatements. The choice of myelographic technique depends on the suspected underlying cause of leak (i.e., dural tear from an osteophyte, leaking meningeal diverticulum, or CSF venous fistula) typically derived from the MRI spine (12). Several myelograms and several treatment sessions may be required to arrive at a diagnosis and cure. An increasing number of negative diagnostics, and lack of response to multiple targeted leak treatments does not exclude SIH from the differential, but should lead the provider to explore additional causes of orthostatic headache, as decribed further below.

### Cervicogenic headache and cervical myofacial pain

Cervicogenic headache represents a heterogeneous collection of disorders, in common representing referred pain to the head. There is no consensus on the single definition of cervicogenic headache so the true prevalence of this condition is unknown (14, 15). The accepted causes of cervicogenic headache are cervical structures innervated by the C1, C2, and C3 nerve roots. Convergence of both nociceptive information from the upper three cervical nerve roots, and the trigeminal nerve at the level of the trigeminal nucleus caudalis in the brainstem is what allows this so called cervicogenic referral of pain to the head (14). The potential sources of pain include: the atlanto-occipital joint, the lateral atlanto-axial joint, the C2 nerve root itself, possibly the C2-3 disc, and the C2-3 facet joint (14). The typical pathologies include degeneration, trauma (for example, whiplash), or inflammation of the respective structure (16).

The osseous portion of the cervical spine is naturally the most mobile segment of the spine in order to allow a wide degree of forward and lateral flexion, extension, and rotation. With this great movement however comes the need for great stability. The spine is stabilized by capsular liagments, the ligaments flavum, the anterior longitudinal ligament, and the posterior longitudinal ligament. The capsular ligaments are extremely strong ligaments wrapped around the facet joints, and serve as the main stabilizers of the spine especially during movement. Cervical instability can result from ligamentous laxity, developing either slowly over time with repeated micro stressors, or from a single load such as a whiplash injury (17). The ligaments do not act alone in stabilizing the spine, the paraspinal musculature plays a significant, albeit secondary role as well. The ligaments contain mechanoreceptors which when stimulated, activate the protective ligamento-muscular reflex to cause a compensatory muscle stiffness (18).

While cervicogenic headache is strictly speaking referred pain from the upper cervical spine, cervical myofacial pain anywhere in the upper or lower neck can refer pain to the head, better termed tension-type headache (16). Cervical myofacial pain of the head and neck, like any myofacial pain syndrome, relies on the formation of trigger points. A trigger point is a defined area of tenderness located within a skeletal muscle, tendon, or ligament. When latent, palpation of the trigger point produces local pain alone but when active, palpation of the trigger point also causes a reproducible referral of pain away from the location of the trigger point (19). While trigger point referral patterns may vary from patient to patient, there are numerous well known trigger point maps identified in humans, many of them responsible for a “cervicogenic” source of headache. For example, pain in the supraorbital or retro-orbital region may be referred from the sternocleidomastoid, the trapezius, or the suboccipital muscles. Likewise, the sternocleidomastoid may also refer pain to the forehead, temple and the vertex. Temple pain may also originate from the trapezius, splenius capitus, suboccipitalis, splenius cervicis, and semispinalis capitis. Occipital pain may originate from the trapezius, levator scapulae, or the semispinalis capitis (19, 20). A comprehensive review of human myofasical trigger points is beyond the scope of this review, however, well-defined human trigger point maps are easily available online (19) and in published literature such as Davies’ The Trigger Point Therapt Workbook (20). Trigger points develop for a variety of reasons, many of them common to hypermobililty. The aforementioned muscle stiffness occuring as a protective reflex to chronic cervical instability is in this author’s opinion the biggest driver. Poor postural habits, lack of exercise, sleep disturbances, malocclusion such as that resulting from temporomandibular joint disease have also been shown to play a role (19). Cervicogenic headaches do not have to be, but can be, orthostatic for a variety of reasons. Upright posture causes an axial loading force, exerted by the weight of the head, on the cervical spine (21). Postures that promote the development of muscle tension and myofacial trigger points, such are head forward posture, are inherent to sitting or standing (22). In the presence of ligamentous cervical instability, it is intuitive that the supine (resting) position naturally reduces exaggerated movement, minimizing strain.

There are no pathogneumonic phenotypes assigned to cervicogenic headache and tension headache referred from the neck in part because this is a collection of multiple disorders. While history and physical exam are helpful in including these etiologies in the differential, the diagnosis may ultimately rest on response to treatment (i.e., a diagnostic and therapeutic procedure). The clinical history that would support cervicogenic headache is of a unilateral pain without side-shift. The pain should be triggered by neck movement, awkward posture, or palpation of the posterior neck. It may be episodic or continuous, and typically moderate in intensity and non-throbbing (14).

The most common cause of a true cervicogenic headache is C2-3 facet joint pathology (spondylosis or capsular strain) (14). The C2-3 facet joint is innervated by the third occipital nerve, a branch of the C3 dorsal ramus (16). The joint itself, located at the level of the mandible, may be tender. Referred pain to the occipital, and less likely the parietal, frontal, and orbital regions may occur. The diagnosis would be confirmed by a third occipital nerve block; a C2-3 facet intra-articular steroid injection or third occipital nerve radiofrequency ablation would serve a therapeutic role (16). Table 2 presents the referral paterns, expected examination findings, diagnostic and therapeutic interventions for the remaining causes of true cervicogenic headache.

**Table 2:** Accepted structural causes of cervicogenic headache (14–16).

Source	Innervation	Referred pain	Physical exam	Diagnosis	Therapy
O-C1 (atlanto-occipital joint)	C2 ventral ramus	Occipital Suboccipital (Temporal)	Pain on head nodding	Atlanto-occipital joint injection	Maximize conservative treatment Atlanto-occipital joint injection
C1-2 (lateral atlanto-axial joint)	C2 ventral ramus	Suboccipital Occipital (Vertex, orbit, ear)	Tender C1-2 joint Restricted passive rotation of C1 on C2 Pain on rotation with a flexed neck	Atlanto-axial joint injection	Atlanto-axial joint injection Atlanto-axial joint RFA Surgical Fusion
C2 nerve root	N/A	Occipital	Associated lacrimation, ciliary injection, rhinorrhea	Selective C2 nerve root block	C2 nerve root injection C2 nerve root pulsed RFA
C2-3 disc	Unknown	Unknown	Unknown	Provocative discography	Fusion
C2-3 facet joint	Third occipital nerve (C3 dorsal ramus)	Upper neck Occipital (Parietal, frontal, orbit)	Tender C2-3 joint	Third occipital nerve block	Third occipital nerve block Third occipital nerve RFA C2-3 facet joint injection

RFA, radiofrequency ablation.

This author’s preference and advice is to thoroughly investigate and treat myofascial sources of referred pain, prior to performing more invasive interventional diagnostics and treatment. For one, myofascial pain often coexists with “true” cervicogenic pathology because of the ligamento-muscular reflex, meaning it is not uncommon to have dual pain generators. Second, myofacial pain is often subject to a self-promoting cascade, in some causes causing more pain then the original joint insult itself. This occurs because sustained muscle contraction reduces the local muscular blood flow, depleting muscle energy reserves, causing more contracticle activity, perpetuating the cycle and eventually triggering inflammation and fibrosis within the surrounding intersitital connective tissues (19). To subject the patient to the least likely to harm interventions first, this author recommends to treat myofacial pain first.

Myofacial cervical pain is often described as pressure-like and dull, but may also feel like throbbing, sharp, stabbing, burning, and heavy. Intensity may range from mild to severe, and pain is usually steady or constant. It may be aggravated by stress, fatigue, chewing, and exercise. It typically has a slow and steady onset, but may occur acutely after a stressful event, trauma, or surgery or dental work particular when awkward postures are sustained. The physical exam may show abnormal posture, and elicitation of a referred pain pattern on examination is key. Myofascial pain is amenable to analgesic medication, topical cold and heat, trigger point massage, trigger point injections, transcutaneous electrical stimulation, and physiotherapy (19). Modifiable factors that lead to the development or perpetuation of myofascial pain should also be addressed including: improper posture, tension, inadequate sleep, and dental malocclusion (19).

### Postural orthostatic tachycardia syndrome

Postural orthostatic tachycardia syndrome is an autonomic disorder most common in child-bearing age females, characterized by orthostatic tachycardia causing but not limited to: dizziness, presyncope, palpitations, fatigue, decreased concentration, tremulousness, and nausea (23, 24). Over half of patients with POTS were found to experience orthostatic headache (24), and POTS is common amongst patients on the hypermobility spectrum (25). The true mechanism of postural headache in POTS has not been well defined. Several mechanisms have been postulated. One thought is that patients develop a relative CSF hypovolemia, either through general hypovolemia or through enhanced spinal absorption. The latter theory implies there is a reduced venous return to the heart in the standing position, resulting in reduced spinal venous pressure, resulting in reduced CSF pressure as CSF is absorbed to the venous circulation. A second thought is that in POTS with severe orthostatic hypotension there is positional ischemia of the posterior neck and shoulder muscles, though this does not account for the variable distribution of head pain in POTS (23). POTS is diagnosed by the finding of a sustained heart rate increment ≥30 beats/min within 10 min of standing or head-up tilt in the absence of orthostatic hypotension (26). The orthostatic headaches of POTS and SIH may be indistinguishable on the basis of associated lightheadedness, fatigue, brain fog, vision blurring, neck pain and nausea. Complicating matters, the headache in both POTS and SIH may repond to volume repletion, abdominal compression (abdominal binder) (23) and temporarily even blood patching [Mokri et al. (23) proposed a mechanism of relative CSF hypovolemia]. POTS should be suspected when the headache is associated with symptoms not typical of SIH or cervicogenic headache including palpitations, tunnel vision, hyperventilation, shortness of breath, or anxiety and/or there are non-headache symptoms that would otherwise be absent in SIH or cervicogenic headache but common in POTS such as post-prandial malaise or onset of syndrome after a viral infection.

The evaluation of suspected POTS should include a detailed history to establish symptoms of orthostatic intolerance, physical examination with postural vital signs to confirm exaggerated tachycardia and absence of orthostatic hypotension, cardiac examination, neurologic examination identifying any features of peripheral neuropathy, laboratory examination with at minimum a complete blood count, electrolytes, thyroid function, and electrocardiogram. Conditions further suspected to be POTS should move on to a 10 min Head-up Tilt Test. The NASA Lean test is another way to easily detect orthostatic intolerance in the clinical setting (27). If small fiber neuropathy is suspected then quantitative sudomotor axon reflex testing and/or testing of epidermal nerve fiber density on skin biopsy is performed (28).

Non-pharmacologic measures build the foundation for treatment of POTS. Medications that worsen sinus tachcyardia such as stimulants or cause hypovolemia such as diuretics should be withdrawn where possible. Blood volume is expanded through a minimum intake of 2–3 L of water per day along with sodium in the form of table salt or electrolyte solutions. Patients are advised to wear abdominal compression binders or high-waist compression stockings to reduce lower body pooling of venous blood. Progressive exercise training to build endurance and increase leg resistance is integral to the treatment plan. The exercise program may initially be recumbent such as rowing, cycling, or swimming, progressing to upright exercise, and patients should be educated that they may initially feel worse. There are no FDA-approved drugs for the treatment of POTS. Pharmacologic therapy is aimed at reducing orthostatic intolerance and symptom management. Fludrocortisone is used as a theoretical blood volume expander. Beta blockers, ivabridine, and pyridostigmine are used to lower heart rate. Midodrine, octreotide, and methylphenidate may be used as vasocontrictors (28).

## Conclusion

Some patients with hypermobility spectrum disorders are uniquely predisposed to orthostatic headaches from a variety of etiologies. In most cases, the clinical history, physical examination, and respective diagnostics should easily distinguish between SIH, cervicogenic headache, tension headache referred from cervical myofacial pain, and POTS. In cases were imaging is negative, using the phenotypic characteristics of the headache disorder is paramount in deciding on the initial treatment pathway.

## Author contributions

OF: Conceptualization, Investigation, Writing – original draft, Writing – review & editing.
